# USING PHOTOVOICE TO EXPLORE THE SALIENCY OF NEIGHBORHOOD LANDMARKS FOR PERSONS LIVING WITH DEMENTIA

**DOI:** 10.1093/geroni/igz038.656

**Published:** 2019-11-08

**Authors:** Kishore Seetharaman, Mardelle Shepley

**Affiliations:** 1 Simon Fraser University, Vancouver, British Columbia, Canada; 2 Cornell University, Ithaca, New York, United States

## Abstract

This study demonstrates the potential of Photovoice, a participatory action research method involving participant-generated photo-elicitation, to explore how persons living with dementia (PLWDs) perceive neighborhood landmarks. Previous research has highlighted the role of well-designed, stable geographical landmarks in improving the navigability of neighborhoods for PLWDs. However, the specific attributes that render landmarks salient have not yet been sufficiently explored, resulting in inadequate evidence-based environmental design guidelines for dementia-friendly communities (DFCs). To address this gap, a Photovoice study was conducted with five community-dwelling PLWDs and their care partners, as part of a dementia-friendly neighborhood walking program in the city of Seattle, USA. Photovoice facilitated the exploration of saliency of neighborhood landmarks from an emic perspective by empowering PLWDs to identify and take photos of salient landmarks during the group walk and interpret and reflect on attributes that contributed to saliency using the photos as visual aids in a focus group discussion and survey questionnaire. PLWDs associated the saliency of landmarks not only with objective physical attributes, e.g., size, shape, color, texture, but also with subjective factors linked to their past, passions, hobbies, and emotions related to having dementia. Findings suggest that the design of outdoor landmarks should satisfy universal design principles, as well as aspects of familiarity, recognizability, and memorability, to ensure that the neighborhood physical environment provides navigational support to PLWDs. The study proposes using Photovoice to facilitate community engagement in the planning and design of DFCs and mobilize people’s lived experience to generate more robust dementia-friendly environmental design guidelines.

